# Seeking tirelessly for better health and life conditions for the child with myelomeningocele*

**DOI:** 10.1590/1518-8345.3957.3428

**Published:** 2021-05-21

**Authors:** Maria Aparecida Bonelli, Amanda Aparecida Borges, Renata Olzon Dionysio de Souza, Gabriela Van Der Zwaan Broekman Castro, Gabriel Brassi Silvestre de Oliveira, Giselle Dupas

**Affiliations:** 1 Universidade Federal de So Carlos, So Carlos, SP, Brazil.; 2 Centro Universitrio Central Paulista, So Carlos, SP, Brazil.; 3 Universidade do Estado de Minas Gerais, Departamento de Enfermagem, Passos, MG, Brazil.; 4 Universidade de Araraquara, Araraquara, SP, Brazil.; 5 Hospital Israelita Albert Einstein, Vila Santa Catarina, So Paulo, SP, Brazil.

**Keywords:** Meningomyelocele, Chronic Disease, Family, Child Care, Nursing, Grounded Theory, Mielomeningocele, Doena Crnica, Famlia, Cuidado da Criana, Enfermagem, Teoria Fundamentada, Meningomielocele, Enfermedad Crnica, Familia, Cuidado del Nio, Enfermera, Teora Fundamentada

## Abstract

**Objective:**

to understand the experience of families in the care of children with myelomeningocele.

**Method:**

a qualitative research study, which adopted Symbolic Interactionism and Grounded Theory as its theoretical and methodological framework, and the semi-structured interview as a data collection instrument. Twenty-eight participants from thirteen families living in a municipality in the inland of So Paulo took part in the study.

**Results:**

the family tirelessly seeks better health and life conditions for the child with myelomeningocele; aiming at the childs autonomy, it is mobilized to the necessary treatments, to learn the care, to adapt the routine, as well as to supply for all the childs needs so that it reaches potential development, looking for a less dependent future with inclusion and social ascension.

**Conclusion:**

family nursing shows potential support in the face of myelomeningocele, due to its ability to apprehend the family system, evaluate it and enable intervention proposals in the care process. In this study, rehabilitation nursing was also emphasized, since it goes together with the child and the family who experience myelomeningocele.

## Abstract (pt)

**Objetivo:**

compreender a experincia de famlias no cuidado de crianas com mielomeningocele. Mtodo: pesquisa qualitativa, que adotou como referencial terico e metodolgico o Interacionismo Simblico e a Teoria Fundamentada nos Dados, e a entrevista semiestruturada como instrumento de coleta de dados. Vinte e oito participantes de treze famlias residentes em um municpio do interior paulista integraram o estudo.

**Resultados:**

a famlia busca incansavelmente por melhores condies de sade e vida para o filho com mielomeningocele; objetivando a autonomia da criana, se mobiliza aos tratamentos necessrios, a aprender os cuidados, a adaptar a rotina, assim como suprir por todas as necessidades da criana a fim de que esta alcance desenvolvimento potencial, prospectando um futuro menos dependente com incluso e ascenso social.

**Concluso:**

a enfermagem familiar mostra-se apoio potencial frente condio da mielomeningocele, devido sua capacidade de apreender o sistema familiar, avali-lo e possibilitar proposies de interveno no processo de cuidado. Neste estudo enfatizou-se tambm a enfermagem de reabilitao, uma vez que esta caminha conjuntamente criana e famlia que experienciam a mielomeningocele.

## Abstract (es)

**Objetivo:**

comprender la experiencia de las familias en el cuidado de nios con mielomeningocele.

**Mtodo:**

investigacin cualitativa, que adopt el Interaccionismo Simblico y la Teora Fundamentada como marco terico y metodolgico, y la entrevista semiestructurada como instrumento de recoleccin de datos. Participaron del estudio 28 participantes de trece familias residentes en una ciudad del interior de So Paulo.

**Resultados:**

la familia busca sin cesar mejores condiciones de vida y salud para el nio con mielomeningocele; con el objetivo de estimular la autonoma del nio, se realizan los tratamientos necesarios, se aprenden los cuidados, se adecua la rutina y se satisfacen todas las necesidades del nio para que alcance su desarrollo potencial, proyectando un futuro menos dependiente con inclusin y ascensin social.

**Conclusin:**

la enfermera familiar muestra un potencial apoyo frente a la condicin de mielomeningocele, debido a su capacidad para aprehender el sistema familiar, evaluarlo y posibilitar propuestas de intervencin en el proceso de atencin. En este estudio tambin se enfatiz la enfermera de rehabilitacin, ya que va de la mano del nio y la familia que padecen mielomeningocele.

## Introduction

Considered the most common congenital malformation of the central nervous system, myelomeningocele (MMC), is characterized by the incorrect closure of the neural tube with exposure of the spinal cord and meninges, during embryonic development^(^ 1 ^-^ 2 ^)^, which may be related to genetic, environmental and maternal factors, especially nutritional deficiency of folic acid, the main risk factor related to this pathology^(^ 2 ^)^.

MMC affects 1 in every thousand births in the world^(^ 3 ^)^ and 1.9 for every 10,000 live births in Brazil^(^ 4 ^)^, which can be identified throughout the vertebral extension, with prevalence in the lumbar, lumbosacral and sacral regions^(^ 2 ^,^ 5 ^)^. This location of the lesion is related to the extent of paralysis, sphincter dysfunction and orthopedic deficiencies^(^ 5 ^)^, as well as intracranial abnormalities, such as hydrocephalus, present in 80% of the MMC cases^(^ 2 ^)^.

Due to the exposure of the spinal cord and meninges, MMC requires surgical repair in the first hours of life, preventing the spillage of cerebrospinal fluid, avoiding infections and decreasing morbidity and mortality^(^ 6 ^)^. Due to the necessary measures and procedures, individuals with MMC require specialized and multidisciplinary health services to increase their functional gains, as well as the support of their families in carrying out their daily activities, given the implications of neurogenic mobility, bladder and intestine^(^ 6 ^-^ 7 ^)^.

The care required by MMC considerably exceeds that required by a child with typical development, and lasts for a lifetime^(^ 8 ^)^, also inferring greater difficulty in psychosocial interaction and lower quality of life in relation to their physical health^(^ 9 ^)^, which generates feelings of fear, insecurity and anxiety in the family, the main social support to ensure the childs growth and potential development^(^ 10 ^)^. Thus, given the repercussion of MMC in the life of the child and his family, it is necessary to get closer to know the familys potentials, weaknesses and rearrangements, understanding the meaning of the care relationships^(^ 11 ^)^, so as to instrumentalize the performance of the nursing professional for qualified care for these families, considering the increase in the life expectancy of this population, and the gap in the literature with this focus^(^ 8 ^,^ 12 ^)^. In this sense, the objective was to understand the experience of families in the care of children with myelomeningocele.

## Method

A field study with a qualitative approach, considering the magnitude of the phenomenon under study, recognizing the subjectivity and intersubjectivity of the relationships^(^ 13 ^)^, which referred to the theoretical perspective of Symbolic Interactionism (SI) and, as methodological framework, the Grounded Theory (GT).

SI, an interpretative reference, recognizes that human behavior is established from social interactions and meanings attributed to objects and actions^(^ 14 ^)^. This framework is potential to understand the familys experience in caring for the child with MMC, considering that individuals interpret their experiences through the meanings learned in the relationships and the social context where they are inserted^(^ 15 ^)^.

When considering the importance and breadth of the families experience in caring for children with MMC, the choice of the GT stands out, which seeks to understand reality from the perception that a certain situation or object has for people, providing knowledge, increasing understanding and generating a significant direction for action^(^ 16 ^)^.

Twenty-eight participants from thirteen families of children diagnosed with MMC living in a city in the inland of So Paulo took part in the study. The concept of child of the Child and Adolescent Statute (Estatuto da Criana e Adolescente, ECA) was adopted^(^ 17 ^)^. The criteria for the selection of participants were the following: a) families of children diagnosed with malformation; b) family members who are able to provide understandable narratives. The participants were identified from the first letter of their kinship: mother (M), father (P, pai in Portuguese), grandmother (A, av in Portuguese) or brother (I, irmo in Portuguese); with the child identified as the index case (C) of the research, followed by the order in which the interview was conducted.

The families were contacted using the snowball technique (snowball sampling), a sampling method highlighted to reach specific groups of people, which, for the initial samples, uses key-informants in order to locate them^(^ 18 ^)^; this trigger occurred in a rehabilitation service in the municipality, with an indication of the first family and, from this, other contacts were revealed, and invitations were made via telephone. The search for families in the advancement of the research was guided by theoretical sampling, assumptions of the GT, which seeks relevant data to develop the theory, being used to substantiate the characteristics of the categories until new elements appear^(^ 16 ^)^; this aspect determined the number of participating families, based on the experiences revealed in the statements, which began to repeat and have the necessary depth for the construction of the theoretical model^(^ 19 ^)^.

Of the four sample groups in the study, the first was made up of four families with the aim of getting closer to the experience, building the initial categories and directing the next groups. From such families, only one had a single caregiver and depended entirely on the health service and public transportation, which leads to reflections on the social vulnerability and support network, which directly influence the experience of caring. Thus, in order to approach families that used the public health service, they were sought in a public institution, contemplating four families that depended on the public health service and articulated a support network for care, composing the second sample group.

These first two groups were made up by school-age children, with a gap emerging from early childhood experiences. Therefore, the third sample group apprehended the particularities of care in the initial phases of the childs growth and development, being composed of three families. The fourth group, composed of two families, validated the theoretical model and, in order to cover as much as possible the families experience, the first validation was carried out with a family whose child was 5 years old, mainly reflecting the intense phase of growth and development, and the second with the family of a 14-year-old adolescent, chosen by the fact that he has already lived all his childhood, thus having an expanded look at the phenomena that make up the experience.

Data collection took place from March to December 2018, through a semi-structured interview with the following guiding question: Tell me how your care trajectory for (childs name) has been? The interviews took place mostly at the familys home, with one at the mothers workplace and another by videoconference, with a mean duration of 69 minutes, in a single meeting. All the family members present at the time of the interview were included in the research, also involving those aged under 18 years old. The number of participants ranged from one to four, with the mother present in all of them, the child with MMC in 6 interviews and the other members involved were father, sister and grandmother.

Data was collected through a digital voice recorder, transcribed in full and analyzed simultaneously according to the GT framework, and the analysis was contemplated by the coding proposed by Strauss and Corbin^(^ 16 ^)^, in three phases: open, axial and selective. The entire route of data collection and analysis was carried out by the first author of this study.

Open coding took place through the process of line-by-line analysis of open codes for the construction of the initial categories; axial coding under reflection of the Paradigm Model; it was structured in five components: causes, intervening conditions, context, strategies and consequences; selective coding, organization stage of the analytical process, allowed identifying the central category of Seeking tirelessly for better health and life conditions for the child with MMC from the theoretical model of the study. This model was presented to two families for validation and constituted the final stage of the GT, important to analyze the pertinence and representativeness of the study in relation to the investigated phenomenon^(^ 20 ^)^.

All the ethical recommendations set forth in Resolution 510/2016 were respected, and the study was approved by the Ethics and Research Committee with Human Beings under CAAE 76493617.3.0000.5504.

## Results

**Figure 1:**
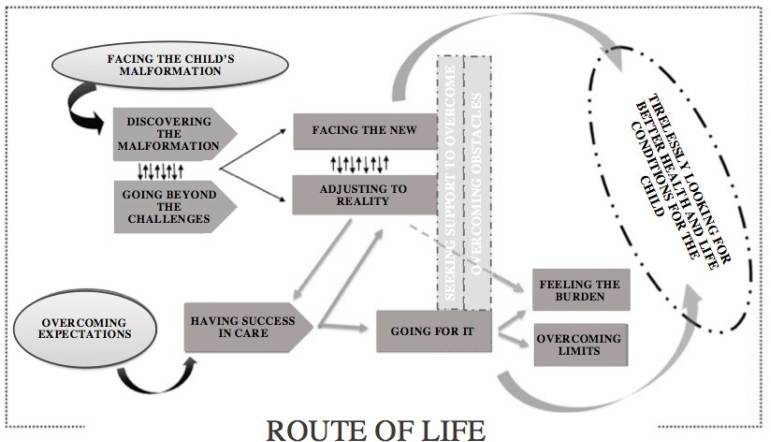
Theoretical model

The childs desire for autonomy and future independence is the center of the experience that drives the family to live a constant process of Seeking tirelessly for better health and life conditions for the child, a theoretical model elaborated in this study, composed of two phenomena, Facing the childs malformation and Exceeding expectations, subdivided into the five components of the Paradigm Model interpreted in the light of SI (Figure 1). It is a complex experience that begins with the diagnosis of the malformation and takes place in a constant process of confrontation and overcoming in different phases of the childs life.

The first phenomenon points out the moments of difficulties experienced by the family with the discovery of the diagnosis; uncertainties and the fear of caring; uncertain prognosis; incessant treatments; mobilization of family members to meet the childs health needs; support received; and obstacles faced. And the second portrays the familys movement beyond curative treatments, through stimuli, devices and physical activity to overcome the childs limitations. They value the childs awareness of the difficulties, participating and enhancing the treatments, in addition to encouraging self-care, prospecting for future independence.

Thus, in order to better explain the core of the whole process experienced by the families, some key elements were highlighted in the course of the lived context, highlighting the data analysis as: Living foggy moments; Going for it and Winning and Thriving.

Living foggy moments is a context of family experience that runs through the entire lived experience. In the diagnosis and birth, they face the malformation, little known or even unknown, with different disorders that can be associated, which darkens the future. This moment is usually accompanied by feelings such as shock, surprise, sadness and fear, and is also related to the way in which the professionals approach and welcome the family in the diagnosis. These feelings are symbols expressed by the families through language in order to elucidate the phase experienced: After I got the diagnosis, I never went back to work, I already left, it was extremely difficult, painful, I came home, locked myself in my room, cried, I didnt want to work anymore, but at the same time, what I was going to be doing here, just thinking nonsense [...] (MF8).

After suffering from knowing and accepting the diagnosis, the family is faced with comorbidities and limitations related to MMC, experiences hospitalizations, complications, various surgeries and uninterrupted treatments, culminating in long recoveries. In addition to the present concerns, there are future fears, surrounded by the fear of new treatments, adolescence, independence, social inclusion and prejudice: At the beginning, he had many consultations, trips, specialists, and there was another question that we had to see, because he was born with two very crooked feet, so we knew that he had already done the valve and the column, but I knew that he had to do the foot, so, it is a lot of agony, but at the same time it was pleasant, because it was a baby, independent with MMC or not, it was my baby who was there, so it wasnt easy, we went to heaven, went down, and we had to go up and down several times (MF1). I worry about how the future will be, how it will develop going forward, and also with society, how it will be treated [...] how will the baby react to that [...] (PF3).

By overcoming the challenges caused by the malformation, the family mobilizes itself to provide better living conditions for the child, offering treatments that allow it to develop in the best way. For this, they overcome financial and emotional obstacles, lack of support from friends and family, inefficiencies of the health services, lack of school inclusion and prejudice: [...] everything costs, the trip to medical appointments costs, and it was every week, a session of physiotherapy costs, and there were two a week [...] We have no financial support from our parents, it was all me and him, we undoubtedly got into debts [...] (MF6). Everyone abandons those who have a child with a problem, its incredible, friends get away, the family disappears, I dont know what they think, they think were going to borrow money, add everyone, I dont know why (PF5). When we started the probe, we had no support from nursing, we felt a lot of difficulty, because the doctors were from outside and they sent us to start the probe, all from the health clinic and we didnt have this here, in fact, we had a fight to get the probe, because we couldnt, only after a long time (MF3). Several schools did not accept, it is difficult, and several schools do not accept children with some type of difficulty [...] (MF9).

Going for it is the familys movement to enhance physical development, autonomy, independence and social inclusion, seeking beyond the childs victory the natural return to the family routine. Acting as a protagonist in this trajectory, through a process of constant symbolic interactions with itself, with each other and with society, the family is influenced by its past experiences, values and beliefs. The desire and need for knowledge and information are continuous, learn to care and encourage, in order to minimize the childs limitations: Each decision made is a set, we talk to the doctors, so, always thinking about what is best, the benefit and we have never made any wrong decisions until now, thank God (MF3). Over time we tried to find out what MMC was, looking for doctors, she started doing physical therapy [...] we always looked for everything they said would be good (MF4). I always tell him that it is not because he does not walk, that he cannot have autonomy [...]. At school, they also encourage independence [...] so we always provide him with autonomy (PF9).

By adapting to the new condition experienced, the family finds itself adjusting to reality, changing the routines of work, leisure and domestic commitments providing all the needs of the child. They seek to adapt the house to promote accessibility and autonomy for the child, in addition to sharing care with the nuclear and extended family, with emphasis on the participation of the father, reducing the burden and providing interaction, bonding and learning between these members and the child: Today our routine is different in the sense of being more racing; to be able to give her what she needs, double work, her father works at night to help me during the day, so our routine changed that. He helps me a lot, with all the care, he stays with her all day, more than I do [...] she has also used catheterization since 1 and a half years old, before it was just me who were doing this, today my mother and her father have learned, so if necessary, one of the three of us goes there, so as not to stop doing all this (MF4). I worked outside, I had to stop, I even tried to work when she was born, because it was always just her and me, it was difficult, I worked a while, but it was tiring, at other times I managed to work part time, the time she stayed at school, but then it didnt work, because she got sick, it was exams, doctors, consultation; get a job and be absent, its complicated (MF7). [...] to make it easier we reformed the house, we removed the steps, we placed wooden carpeting, because we knew that she was always on the floor, we always tried to do things to make her feel as independent as possible (PF3).

The family is strengthened, seeking support for overcoming, valuing all the attention received, being beyond family and friends, also involving the support from the health institutions/professionals, school and spirituality that provides support to face the situation: [...] my father-in-law took care of him, took him to physiotherapy, everywhere, and I went to work, since then I never stopped. He really did help us (PF5). [...] regarding the care I have to have with him, in relation to guidance, necessary materials, in relation to living together, I am grateful to the Health Unit, I feel very embraced there (MF1). Faith has strengthened me in everything, it is my basis, my prayers, because it is a constant concern for it [...] The school is adapted, and some more adaptations were made for the caregiver to make the change, she is the first wheelchair student, and they adapted some things, such as the mat, a few little things (MF7).

All these family strategies result from the individual and relational actions of its members, which mobilize the family system, acting cooperatively to solve the problems. These interactions allow the individuals to put themselves in the others place and, through symbolic communication, understand the symbols expressed, defining their actions together with the others, acting collectively, articulating strategies that help in solving the childs real needs and of the family.

Winning and Thriving goes beyond the results achieved by the family in the trajectory, as well as it permeates all future anxieties. The familys route is accompanied by the desire for autonomy and future independence, which, as they are achieved, provide comfort and satisfaction, in addition to stimulating them to continue the struggle, to thrive again. Dreams and expectations permeate all the family relationships, the desire to see the child win is the greatest of them, as well as the longing for him to accomplish everything he wants: So, each of his conquests for me, is something out of the ordinary, it is different from everything I have ever experienced, he with his limitations, is in the middle of 99 children who have nothing, interacting equally, I speak like this, that for me, Im enjoying every second from when I received it (MF1). Last year there were the Olympics at school, everyone applauded the child, I even cried, he did the exercise with the basketball just like all the kids, he hit the ball with one hand, and with the other he pushed the chair, everyone clapped their hands, that for me was wonderful (MF9). People say, retire her, and I say that this is not my decision, I say that I want her to be independent, to study, to have her life, not that she does not depend on anyone, even with her limitation, but that she may have the autonomy to live her life (MF4).

Upon perceiving the child developing and overcoming limitations, the family, despite the arduous and incessant battle, feels their effort producing results, which makes them value the experience even more, feeling the winner in each obstacle overcome, re-signifying life from then on: She came and turned everything upside down, but all for the better, for the very best, I am very happy and very grateful to God for giving me this gift, because it did me so well, my greatest happiness [...] (MF2).

The family renews itself every day for what is to come, always planning, foreseeing, anticipating, learning and doing everything for the child to develop and progress. Care becomes less intense, it is characterized as a natural routine; tension and tiredness do not stand out from the joy of seeing the child overcoming his limits, winning victories, which propels the family into the future, overcoming limits: You know, things ended up getting so normal, I dont even think its care, they happened so naturally, even the probe ended up being natural. She does everything she wants, dances, sings, does not stop [...] (MF2). We program ourselves on what she needs [...], we always say that our life is normal so fast that it was the adaptation of our routine in relation to her [...] if we go to the beach, we look for a more peaceful beach [...], we will always look for a place that will also be good for her [...] and we reprimand them when we have to punish them when it is really due (PF3).

## Discussion

The empirical analysis of the data allowed for the apprehension of the theoretical model of Seeking tirelessly for better health and life conditions for the child, which represents the meaning of family actions and interactions in the experience of caring for children with MMC.

In this family trajectory, the childs desire for potential development is highlighted, with autonomy and social ascension, mainly related to preserving the cognitive functions. The family seeks the contribution of the health professionals to provide care and rehabilitation. In this perspective, rehabilitation nursing, which values family-centered care, highlighting their different needs and recognizing their individualities^(^ 21 ^)^, becomes promising, but little explored in the route of these children.

The diagnosis is a trigger for family experience, surrounded by sadness, insecurity and shock at the unexpected. The obstacles of this occasion are intensified by the inadequate approach of the professionals^(^ 7 ^,^ 10 ^,^ 22 ^-^ 24 ^)^. The constant interaction with health professionals who have superficial information, little empathy and no welcoming attitude, generates distrust, doubt, fear and helplessness. There is little clarity in the information, with emphasis on the different afflictions associated with MMC, such as type of injury, treatments and response to the therapies. In this context, the experiences are different and the family members build their actions and meanings through a process of symbolic interaction with themselves and with the others^(^ 25 ^)^.

The child with MMC has comorbidities and limitations that change with growth and development, which causes the trajectory to be surrounded by treatments, hospitalizations, arduous recoveries and distancing from social and school life^(^ 8 ^)^. There is suffering intrinsic to the confrontations, intensified by the complications experienced, in the face of infections, surgical approaches and uncertainty for the recoveries. The meaning attributed to the experiences influences and directs the way individuals act^(^ 14 ^)^.

The chronic condition imposed by MMC demands specialized care, which requires direct and continuous supervision by health professionals^(^ 12 ^,^ 26 ^-^ 27 ^)^, so as to maintain functionality, prevent harms and promote autonomy. A literature review portrays the relationship between the potential reach of better health conditions in individuals with MMC and assistance in specialized centers, with this discrepancy also associated with ethnicity and social condition^(^ 28 ^)^, so the lack of health services aimed at rehabilitation becomes an impasse in the care of children with MMC^(^ 10 ^)^, above all, with regard to the transition to self-care, as observed in this study.

The promotion of care for MMC and comorbidities, demands professional guidance, potential field of nursing, considering the specificity of procedures such as dressings, intestinal lavage and bladder catheterization. When the health team exercises effective support, it can mean a base for the family, where it builds its relationships in favor of child care, allowing it to invest in their development^(^ 12 ^,^ 29 ^)^, which however proved to be incipient in this study.

During this experience, the family experiences numerous deprivations and routine changes, physical and mental exhaustion, financial overload, lack of support from friends and family, which lead to social isolation^(^ 10 ^,^ 24 ^,^ 29 ^-^ 30 ^)^, adding prejudice, an important aggravating factor in this experience, as well as in the lives of other families of children with special health needs^(^ 31 ^-^ 32 ^)^.

A study developed with mothers of children with MMC showed the economic burden, difficulty in accessing specialized services, school and transportation, as well as the need to adapt and/or move to another residence^(^ 22 ^)^. The need to purchase supplies for care and support and mobility devices, when the supply of the public health service is insufficient, increases the financial burden, psychosocial issues and changes family functioning; this reality is also portrayed in other studies of families of children with MMC and other chronic health conditions^(^ 8 ^,^ 24 ^)^.

Another aggravating fact which emerges is that mothers, when taking care of a child with a chronic condition, quit their jobs, due to the difficulty of the duality of the routine^(^ 8 ^,^ 33 ^)^. Giving up professional life was also a behavior observed in this study, where five mothers, feeling overwhelmed with the excess of functions attributed to the chronic disease, left their professional life. This behavior was not adopted by most of the participants, who, strengthened by the support received, managed to maintain their employment contract, which allowed for interactions in extra-family environments, reducing the emotional exhaustion resulting from the uninterrupted immersion in the childs care routine.

Although a number of studies demonstrate the role of primary caregiver assigned to the mother^(^ 10 ^,^ 12 ^,^ 33 ^-^ 34 ^)^, this study showed, in a predominant manner, that in families where the father was present in the family nucleus, he also exercised the role of caregiver, sharing care and enhancing the family bond. The fathers participation has been evidenced in the life and care of the child with special health needs through daily care, stimulating the bond and affection of the relationships between them^(^ 35 ^-^ 36 ^)^; caring for the child in a shared way improves family interactions^(^ 36 ^-^ 37 ^)^.

For the family, relating to people other than the family core is important. Some health professionals, more distant family members and friends provide significant support. The relationship with peers, that is, parents who have children in the same health condition, promotes a feeling of welcoming and belonging to society^(^ 38 ^)^.

Spirituality, support strategy and coping in the route of young people with a chronic disease and their families^(^ 39 ^)^, was praised by the participants, from the diagnosis to all the experienced stages. Faith is considered a vital element in the search for meaning in the face of the malformation; the belief that it is Gods will and/or that God believed in the strength of the family to take care of this child, mobilizes members in the face of difficulties. For the health team, recognizing the importance of spirituality as a support resource allows creating strategies to strengthen this family^(^ 40 ^)^.

On the other hand, over time, the family moves towards adapting to the new routine, striving to insert the child into routine and leisure activities, seeking to act normally and feel part of society^(^ 29 ^)^. Thus, they impetuously desire that the child, despite their limitations, gains autonomy and social insertion^(^ 41 ^)^; standing out as a strategy for this purpose is physical activity, which, in addition to health benefits and functional development, promotes independence, motivation and social participation^(^ 42 ^)^, such experiences also being apprehended in this study.

Regarding the perception of children and adolescents towards MMC, a study developed in Ireland shows that they are self-confident in the face of their disability, with good social and school participation^(^ 23 ^)^. Children participating in this research report on their participations, school interactions, sports and leisure, highlighting joy and enthusiasm in their social relationships and achievements.

In the context of the chronic health condition, in this case, MMC, socialization is a preponderant yearning throughout all the phases of growth and development, considered essential for the childs future. In a literature review, social participation and interaction is identified as an important strategy in the rehabilitation process, and its promotion through the available support network^(^ 43 ^)^, an action that is apprehended in the families reports, which moves very hard to overcome limitations and acquire motor, cognitive and social skills.

The way the family settles and involves the child in the interactional processes inside and outside the family environment interferes with the childs developmental capacity for the emission and interpretation of symbols, interactions and decision-making. Caring in all the growth and development phases, linked to unconditional love relationships, affection and attention given by the family, is essential for the child to reach its maximum potential in the life trajectory.

Nursing, despite its historical role in the art of caring, was not evidenced in the experience of these families. Thus, with the development of this model, we seek to provide nurses with subsidies to work with the family of the child with MMC. Considering the diversity of performance of the professional nurse, the highlight was for rehabilitation nursing, evidenced in the experiences, given the family members desire to be trained to care and promote the childs autonomy. Likewise, family nursing also proves to be a potential support due to its ability to apprehend the family system, evaluate it and enable intervention proposals in the care process. Thus, the study makes an important contribution, the role of the father in the care of the child with MMC, highlighting the esteem of understanding family interactions and their potential.

As for the limitations, the fact that the study was carried out with a specific age group stands out, which points to other phenomena to be explored, and that the municipality is not a reference for treating MMC, interfering in the process of self-care and independence for the upcoming development phases. It is noteworthy that the portrayed experience does not generalize that lived by all the families, as always something new can emerge and be deepened for later inclusion in the theoretical model, characteristic of the method employed.

## Conclusion

The central category denotes that the ability to love unconditionally drives the family in the face of the desire to provide better chances for the child to grow up, to develop, to be inserted in society and to be respected in view of the place reached in relation to his qualities and abilities, that Being a child stands out from the difficulties faced. Care may come, in addition to promoting child development, to minimize situations where it does not occur as expected, such as MMC; however, this task needs to be expanded beyond the family members, shared with the health services, making necessary resources available to the child, promoting health and reaching their physical, cognitive, emotional and social potentials.
